# How was the proton transfer process in bis-3, 6-(2- benzoxazolyl)-pyrocatechol, single or double proton transfer?

**DOI:** 10.1038/srep25568

**Published:** 2016-05-09

**Authors:** Yongjia Zhang, Mengtao Sun, Yongqing Li

**Affiliations:** 1 Department of Physics, Liaoning University, Shenyang 110036, P. R. China; 2 Beijing National Laboratory for Condensed Matter Physics, Beijing Key Laboratory for Nanomaterials and Nanodevices, Institute of Physics, Chinese Academy of Science, Beijing, 100190, P. R. China

## Abstract

A theoretical analysis of proton transfer process for the symmetric systems with two intramolecular hydrogen bonds, bis-3,6-(2-benzoxazolyl)-pyrocatechol(BBPC) in hexane solvent, has been researched. In this study, we utilized ωB97X-D/ 6-311 + g (d,p) and B3LYP/6-31 + G(d) two procedures calculating the foremost bond length and bond angle, respectively. Our calculations demonstrate the two intramolecular hydrogen bonds were strengthened in S1 state, thus the proton transfer reaction can be facilitated. Furthermore, the calculated IR vibrational spectra confirmed hydrogen bonds were enhanced in S1 state. We found three local minima A B and C from the potential energy surfaces (PESs) on the S1 state, and the energy of B point and C point are identical. A new ESIPT mechanism has been proposed that was not equal to the previous conclusions. The new ESIPT mechanism elucidates that single proton transfer more likely occurs in the symmetric BBPC molecule in comparison with the double proton transfer reaction. And the frontier molecular orbitals(MOs) further illustrate the trend of ESIPT reaction.

Owing to the importance of hydrogen bond playing in nature, it has been researched and reported numerous publications on the relevant topics in the past few years. Particularly, Han and his partners put a new theory to explain the dynamic of excited-state hydrogen bonds^1^,^2^,^3^,^4^,^5^,^6^,^7^. The novel mechanism can well clarify some chemosensors via the interaction of inter- and intra- molecular hydrogen bonds, such as intramolecular charge transfer (ICT), excited state proton transfer (ESPT), photoinduced electron transfer (PET), fluorescence quenching, photoinduced electron transfer (PET), and so on^8^,^9^,^10^,^11^,^12^,^13^,^14^,^15^,^16^,^17^,^18^,^19^,^20^. The excited state intramolecular proton transfer (ESIPT) reaction belongs to the essential unimolecular processes in nature, as one of the fastest and quit complex processes in nature and now has been studied by several modern ultrafast technology. ESIPT reaction has been a popular research projects of photochemistry and photophysics up to now since 1956, firstly investigated by Weller *et al.* with the particular experiment of methylsalicylate^21^,^22^. The transferred tautomerization leads to strong and fast charge distribution restructuring, these molecules which with the characteristic are very fascinating towards the layout and application of fluorescence chemosensors, laser dyes and LEDs, ultraviolet ray (UV) filters and photostabilizers^23^,^24^,^25^,^26^. More and more spectroscopic techniques have been used to investigate ESIPT reaction in recent years. Even though the enormous volume of endeavor has been dedicated^27^,^28^,^29^,^30^,^31^,^32^,^33^,^34^,^35^,^36^,^37^,^38^,^39^,^40^, the investigations of ESIPT process still remains immense challenges primarily due to the intrinsic complicated processes of physical and chemical property, such as quantum nature, cleavage and formation of hydrogen bond, the change of the excited-state hydrogen bond, nuclear rearrangement process and so forth.

In recent years, a large number of ESIPT reactions literatures discussed the so-called double benzoxazoles containing two protons^34^,^35^,^36^,^37^,^38^,^39^,^40^. The best-known examples, bis-3,6-(2-benzoxazolyl)-pyrocatechol (BBPC) and bis-2, 5-(2-benzoxazolyl)-hydroquinon (BBHQ), are symmetrical system with double intramolecular hydrogen bonds. Both of them were published by Mordzinski *et al.* firstly, through the nodal plane model method^34^. Their calculation predict that the BBHQ undergoes single proton transfer process in S_1_ state, while BBPC do double proton transfer reaction. On the basis of vibronic spectra and wavepacket dynamics, Weiβ *et al.* considered that an existent double proton transfer process of BBHQ in the excited state^35^. How was the proton transfer of BBHQ, this problem caused many attention of researchers. In order to solve the problem, Zhao *et al.* put forward a new mechanism of BBHQ via the TDDFT method as the basic theory, that the BBHQ undergoes double proton transfer or consecutive single transfer reaction^36^. By contrast, the researches on the proton transfer mechanism in BBPC are very limited. Grabowska and his partners claimed that BBPC exist double proton transfer reaction in S_1_ state^37^. However, Wortmann *et al.* confirmed the single proton transfer happens in S_1_ state of BBPC, which was confirmed by the absorption and emission electrooptical spectra^38^. They proposed that the electrooptical measurements and quantum chemical calculations were the reliable tools for studying the mechanism of phototautomer. Whether single or double proton transfer undergoes in BBPC is worth to be revised.

In spite of the nodal plane model has been proved in a lot of former achievements^42^,^43^,^44^, the proton transfer mechanism perhaps may not be inferred from it merely. It is difficult to detect whether one proton transfers firstly, the second proton transfers subsequently according to the nodal plane model. Unsatisfactorily, we can only get some information about geometric configuration and physical properties of molecular via the spectrum technology. In this work, a theoretical calculation has been implemented to investigate ESIPT reaction mechanism of BBPC. In order to clarify the ESIPT process mechanism of BBPC in detail, DFT and TDDFT methods have been adopted to the calculations for ground and excited state, respectively. Herein, the optimization of geometric structures in S_0_ and S_1_ states have been done, vertical excitation energy were calculated, IR vibrational spectra, the frontier molecular orbitals (LUMOs and HOMOs) as well as the potential energy surfaces (PESs) in the S_0_ and S_1_ states were calculated in our investigation, we choose both ωB97X-D/ 6-311 + g (d,p) and B3LYP/6-31 + G(d) to calculate the foremost bond length and bond angle, so that ensure the accuracy of the calculation result. Similar calculation results were obtained by the two procedures and considering the practical factors only B3LYP/6-31 + G(d) method done the follow work.

Our paper is logical as follows part introduces the calculation of the details. Part3 describes the results and discussion, and by considering the geometry, electronic spectra and the potential energy surface. The last part summarizes the conclusions of the research.

## Results

### Geometric Structures of BBPC

**Figure 1:**
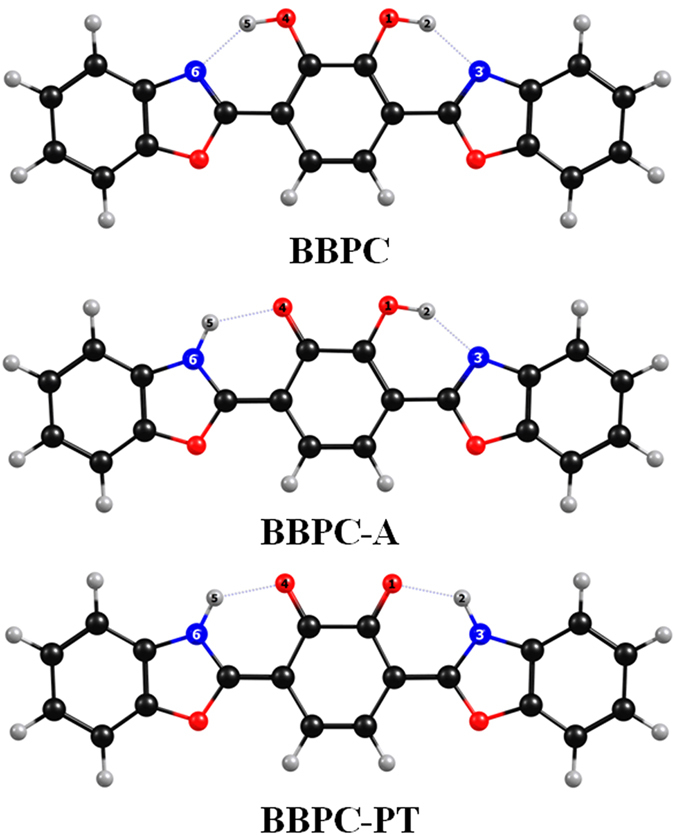
Optimized structures of BBPC, BBPC-A and the BBPC-PT at the B3LYP/6-31 + G(d)/ IEF-PCM (benzene) theoretical level. Red: O; Gray: H; Blue: N; Black: C.

The DFT and TDDFT methods have been adopted to optimize the structures for BBPC in S_0_ state and S_1_ state, respectively. To test for the optimization of the structure is the most stable configuration, we also analyzed the frequency. Considering the solvent effects, hexane has been selected as the reaction solvent in the IEFPCM model. There are three structures of stable isomers BBPC, BBPC-A and BBPC-PT calculated display in Fig. 1. To make sure the description of bond lengths and bond angles more clearly and concisely, the hydrogen bonded atoms have been numbered. Herein, BBPC-A is the single proton transfer form, BBPC-PT is the double proton transfer construction. We listed the most important structural parameters in Table 1, which were calculated by the B3LYP/ 6-31 + G (d) procedure that is related to the hydrogen bonds. Based on our calculated results, it’s worth noting that the bond lengths of O_1_-H_2_, H_2_-N_3_, O_4_-H_5_ and H_5_-N_6_ of BBPC structure are 0.99 Ǻ, 1.81 Ǻ, 0.99 Ǻ and 1.81 Ǻ in the ground (S_0_) state, respectively. However, after being excited to S_1_ state, the bond lengths are 1.01 Ǻ, 1.71 Ǻ, 1.01 Ǻ and 1.71 Ǻ, respectively. Meanwhile, the O-H-N bond angle varies from 145.1° in S_0_ state to 148.1° in the S_1_ state. For comparison, the bond lengths of H_2_-N_3_ and H_5_-N_6_ of BBPC are shorten from 1.80 Ǻ in the ground (S_0_) state to 1.68 Ǻ in the S_1_ state by ωB97X-D/ 6-311 + g (d,p). Significantly, both methods show that the bond lengths of O_1_-H_2_ and O_4_-H_5_ are longer as well as H_2_-N_3_ and H_5_-N_6_ are shorten in the excited S_1_ state, which indicates these two intramolecular hydrogen bonds are simultaneously enhanced in the excited S_1_ state. For BBPC-A structure, the bond length of H_2_-N_3_ decreased from 1.80 Ǻ in the ground (S_0_) state to 1.77 Ǻ in the excited S_1_ state and the concomitant enlargement of O_1_-H_2_-N_3_ bond angle from 145.1°–147.0°. It indicated that the intramolecular hydrogen bond O_1_-H_2_···N_3_ is more stable in the S_1_ state than that in the ground (S_0_) state. Moreover, for BBPC-PT, the double proton transferred form can not appear stable structure in S_0_ state and the subsequent part of the potential surfaces will mention it.

**Figure 2:**
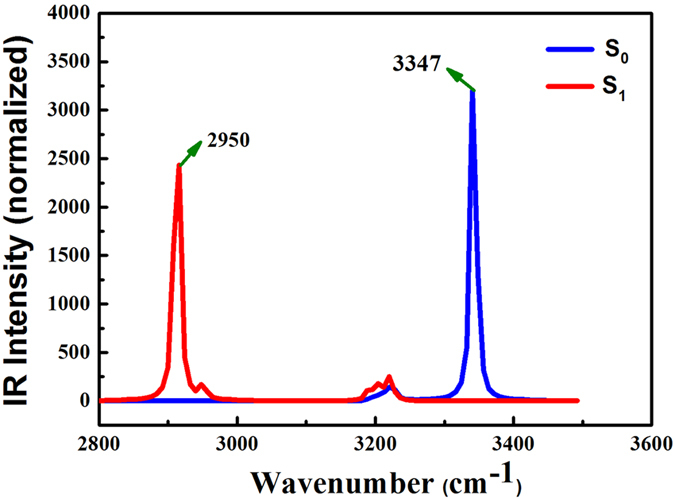
Calculated IR spectra of BBPC in the spectral region of both O-H stretching bands in the S_0_ and S_1_ states based on the B3LYP/6-31 + G(d)/IEF-PCM (hexane) theoretical level.

By observing and analysising the infrared vibration spectrum of related chemical bonds, it can be further used to indicate the hydrogen bond in excited state is different from ground state^1^,^2^,^3^,^4^,^5^,^6^,^7^. Figure 2 provided the O-H stretching vibrational modes of BBPC by B3LYP/6-31 + G(d). Our calculated results for comparison are computed via ωB97X-D/ 6-311 + g (d,p) method, and the calculated stretching vibrational frequency of O-H is around 3469 cm^−1^ in S_0_ state and shift to 2983 cm^−1^ in S_1_ state. As the calculated O-H stretching vibrational frequency using B3LYP/6-31 + G(d) method is 3347 cm^−1^ in the S_0_ state, nevertheless it changes to be 2950 cm^−1^ in the S_1_ state, which are very close to the calculated results, using ωB97X-D/ 6-311 + g (d,p) method. This implies that the hydrogen bonds are reinforced in S_1_ state.

### Calculated Frontier Molecular Orbitals (MOs) and Electronic Spectra of BBPC

**Figure 3:**
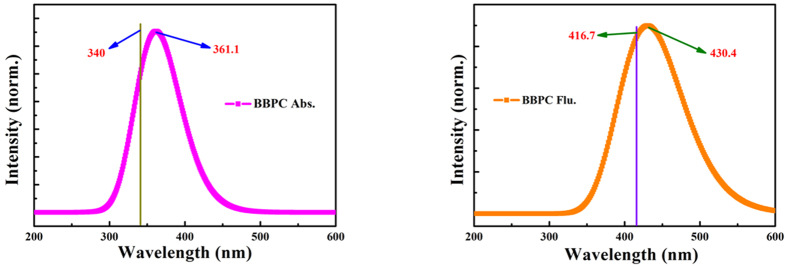
The calculated absorption and fluorescence spectra of BBPC and BBPC-A forms at the B3LYP/6-31 + G(d)/IEF-PCM (hexane) theoretical level. The croci vertical lines show the experimental results.

The electronic absorption spectra, calculated using TDDFT/B3LYP/6-31 + G (d) theoretical method and the IEF-PCM solvent model, have been shown in Fig. 3 with the range of λ = 200~600 nm. The absorption peak and emission peak of BBPC are at 361.1 nm and 430.4 nm, respectively^48^. We can clear see our calculated results has very constant with the experimental results from Fig. 1. Besides, the value only 30.8 nm and 50.1 nm bigger than the results obtained from TDDFT /ωB97X-D/6-311 + g (d,p), respectively, the TDDFT/ωB97X-D/6-311 + g (d,p) results are 330.3 nm and 380.3 nm.Therefore, it demonstrates that the TDDFT/B3LYP/6-31 + G (d) method is feasible and satisfactory. We have tested the two programs and found them that they produce essentially same results in our case. Therefore, we only used the first method. Moreover, the emission peak of BBPC-A is around 521.6 nm, and the 564.1 nm fluorescence hump of BBPC-PT sturcture was also found based on our calculated method.

**Figure 4:**
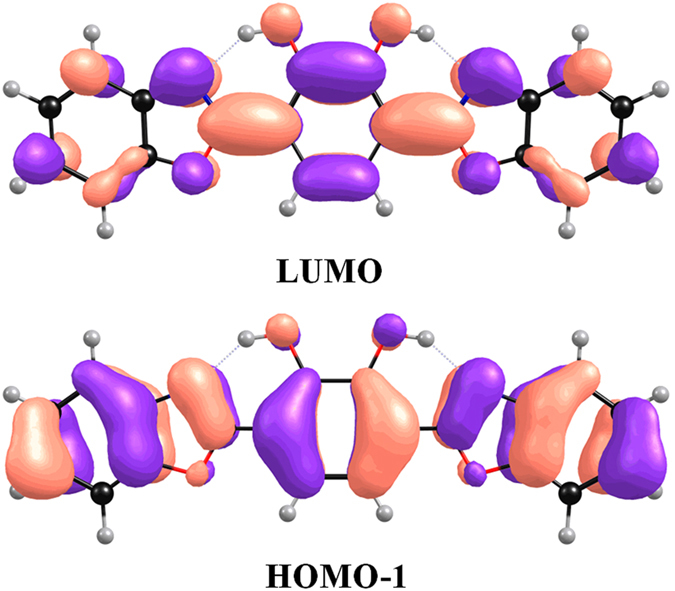
Frontier molecular orbitals, HOMO-1 and LUMO, for the BBPC chromophore based on TDDFT/B3LYP/6-31 + G(d)/IEF-PCM (hexane) calculations.

Figure 4 shows MOs of BBPC in the solvent of hexane. Before we discuss the proton tranfer mechanism in S_1_ state, it’s should be qualitatively analysis the nature of charge distribution and charge transfer. We only display HOMO-1 and LUMO orbitals in Fig. 4 that was because mainly involves the two orbitals in the first excited state(HOMO-1 → LUMO: 97.51%). Obviously, it can be seen that HOMO-1show the π character yet π^*^ character for LUMO, which is defined that the S_1_ state is attributed to the evident ππ* feature. It is worth to pay attention that the electron distribution of HOMO-1 and LUMO orbit for BBPC molecule are different. From HOMO-1 transfer to LUMO, the charge density of hydroxyl moiety were decreased yet for N atom was increased. Moreover, the increased charge density of N atoms make the hydrogen bond enhanced and promoting the ESIPT process.

### Potential Energy Surfaces (PESs)

**Figure 5:**
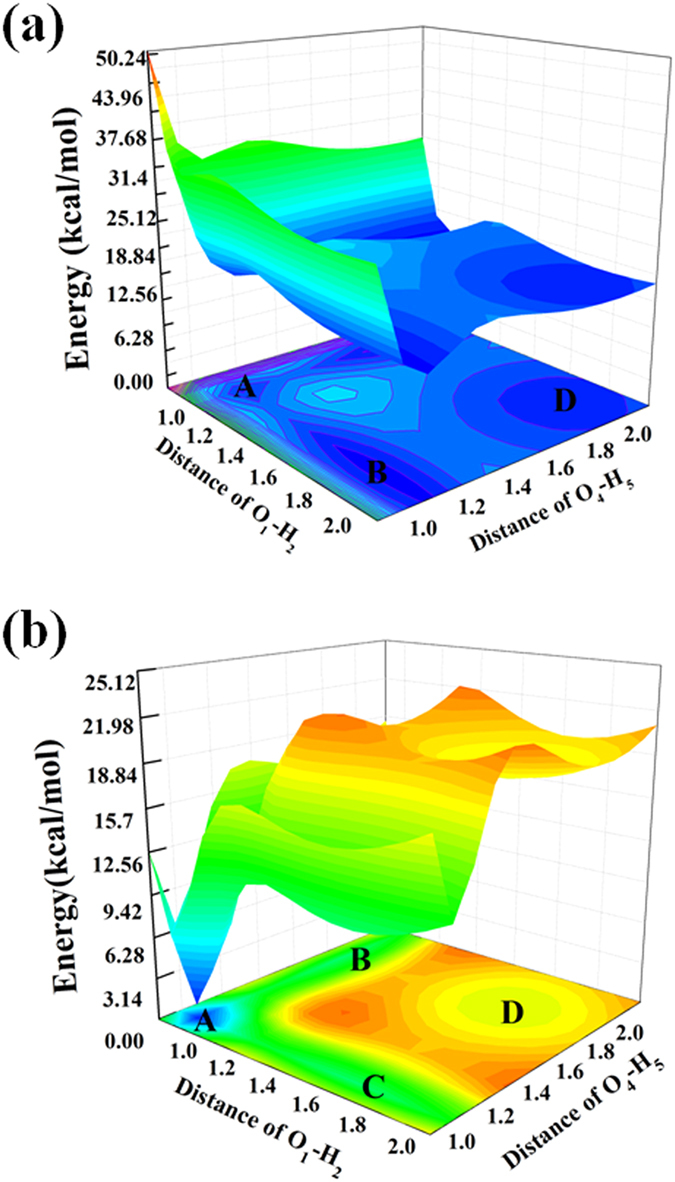
PESs of the S_0_ and S_1_ states of BBPC as functions of the O_1_-H_2_ and O_4_-H_5_ lengths ranging from 0.81–2.11 Å in the S_1_ state and 0.89–2.19 Å in the S_0_ state (**a**) S_1_ state PES; (**b**) S_0_ state PES.

Construction of potential energy surface is an effective method to researching the molecular properties and reactions, and it can be more intuitive understanding the intramolecular proton transfer reaction process. Therefore, the calculation of potential energy surfaces are necessary to clarifying the process of the ESIPT in the BBPC. Potential energy surfaces are using the constrained optimizations in the relative electronic states along with fixing the O_1_-H_2_ and O_4_-H_5_ distances in a serious of values, respectively. The bond lengths of O_1_-H_2_ and O_4_-H_5_ are fixed in the S_0_ state and S_1_ state geometrical structures. Even though the DFT/TDDFT method may not be fully accurate to reproduce the proton transfer process from the surfaces,former works has proved this method to qualitatively analyze the status of excitation energy surface is very reliable and effective, that can provide the proton transfer process with precise proton transfer path^45^,^46^,^47^. The constructed PESs of the S_0_ and S_1_ state of the O_1_-H_2_ and O_4_-H_5_ bond lengths (vary from 0.81–2.11 Å in the S_1_ state and 0.89–2.19 Å in the S_0_ state) are in Fig. 5. The symmetrical PES of the S_1_ state has been displayed in Fig. 5(a) with minima signed. The coordinates are: A (1.01 Å, 1.01 Å), B (1.91 Å, 1.01 Å), C (1.01 Å, 1.91 Å) and D (1.91 Å, 1.91 Å). Herein, the energy of B and C points are the same and symmetrical by the diagonal AD. From the calculated results, we know that the BBPC-A is the most firm structure of the S_1_ state in these minimum potential energies, the relationship are E_A_ > E_D_ > E_B_ (E_C_).

The minimum potential energies are given in Table 2, calculated by B3LYP/6-31 + G (d), for the sake of clarifying the potential energies definitely. Meanwhile, we also calculated the potential barriers among these minimum energy points, the results show that: there is 3.24kcal/mol potential barrier between A and B (C); the potential barrier between A and D is 8.28 kcal/mol; from B (C) need to cross the 6.66 kcal/mol barrier to reach D point. Furthermore, after the radiative transition, single proton forming B (C) structure is the most stable of the three points due to the barrierless process that has shown in Fig. 5(b). Although the potential barrier separates point B (C) from point D is not large, only single proton transfer reaction more likely happens in the S_1_ state. Consequently, we summarized the ESIPT process in BBPC as followed: BBPC exists in the S_0_ state, after the photoexcited to the S_1_ state, it changed be the structure at point A. Then a proton transfers along the hydrogen bond from hydroxyl O to the N atom formed BBPC-A structure.

## Discussion

Summing up, theoretical to investigate the proton transfer reaction process of BBPC chemosensor has been performed by quantum chemical method and IEF-PCM to evaluate the solvent effect. Through analyse the calculated result, the variable bond length, angles and IR vibrational spectra, it can be illustrate that the hydrogen bond enhanced in S_1_ state and the ESIPT process be facilitated. The calculated MOs of BBPC also support the proton transfer in S_1_ state. In order to exhaustive explain, we also calculated PESs of the S_0_ state and S_1_ state, based on constrained optimizations in keeping the O_1_-H_2_ and O_4_-H_5_ distances fixed in a serious of values. There are three local minimum in S_1_ state, which the energy of B point and C point are identically. According to the calculated potential barriers among the minimum points in S_1_ state, single proton transfers via the hydrogen bond.

A new ESIPT mechanism has been proposed that was not equal to the previous conclusions^41^. The new ESIPT mechanism elucidates that single proton transfer more likely occurs in symmetric molecule of BBPC is essentially two equivalent single proton transfer reaction in comparison with the mechanism of double proton transfer.

## Methods

In this study, the S_0_ and S_1_ state configuration of BBPC molecule was optimized by the DFT and TDDFT method, respectively. The B3LYP functional, and the 6-31 + G (d) basis set was used in both the DFT and TDDFT methods^48^,^49^,^50^. The ωB97X-D functional, has satisfactory accuracy for thermochemistry, kinetics, and non-covalent interactions^51^. Therefore, we also calculated based on ωB97X-D functional and 6-311 + g (d,p) basis compared with the B3LYP functional^51^,^52^,^53^,^54^. Combining the integral equation formalism variant (IEFPCM) and the model Polarizable Continuum Model (PCM)^55^,^56^,^57^,^58^, the calculations were performed in hexane solvent. The geometric optimization of the atoms, bonds and angles with no constraints. After analysis of the calculated vibration, all the local minima were demonstrated that has none of an imaginary mode. All theoretical calculations were performed by the Gaussian 09^59^.

The potential energy surface of BBPC in S_0_ and S_1_ state were constructed with the fixing the distance of O-H at a series of values. Secondly, the O-H bond length in S_0_ state variable range is from 0.89 Å to 2.19 Å and changed from 0.81 Å to 2.11 Å in S1 state. In S_0_ state and S_1_ state configuration optimization process, the self-consistent field (SCF) convergence criteria we adopt the default settings 10^−6^ both in S_0_ and S_1_ state. The harmonic vibrational frequencies were determined by the Hessian diagonalization of 60, and the infrared vibration intensity was determined by dipole moments^61^. The excited-state Hessian matrix is based on the analytical gradients of numerical difference and the method we adopted is the central difference method, set the step length as 0.02 Bohr.

## Additional Information

**How to cite this article**: Zhang, Y. *et al.* How was the proton transfer process in bis-3, 6-(2-benzoxazolyl)-pyrocatechol, single or double proton transfer? *Sci. Rep.* **6**, 25568; doi: 10.1038/srep25568 (2016).

## Figures and Tables

**Table 1:** The calculated bond lengths (Å) and bond angles (°) of BBPC in the S_0_ and S_1_ states.

	BBPC		BBPC-A	
	S_0_	S_1_	S_0_	S_1_
O_1_-H_2_	0.99	1.01	0.99	1.00
H_2_-N_3_	1.81	1.71	1.80	1.77
O_4_-H_5_	0.99	1.01	1.77	1.90
H_5_-N_6_	1.81	1.71	1.04	1.03
δ(O_1_-H_2_-N_3_)	145.1°	148.1°	145.1°	147.0°
δ(O_4_-H_5_-N_6_)	145.1°	148.1°	131.0°	127.0°

**Table 2:** The potential energies (Hartree) of stable structures on PESs of the S_0_ state the S_1_ state for BBPC, BBPC-A and BBPC-PT.

	BBPC		BBPC-A		BBPC-PT	
Energy	S_0_	S_1_	S_0_	S_1_	S_0_	S_1_
	−1179.828	−1179.714	−1179.813	−1179.719	−1179.801	−1179.715
